# Microsatellite Loci in the Gypsophyte *Lepidium subulatum* (Brassicaceae), and Transferability to Other *Lepidieae*

**DOI:** 10.3390/ijms130911861

**Published:** 2012-09-19

**Authors:** María Isabel Martínez-Nieto, María Encarna Merlo, Juan Francisco Mota, Esteban Salmerón-Sánchez, José Gabriel Segarra-Moragues

**Affiliations:** 1 Department of Plant Biology and Ecology, CITE-2, University of Almería, Carretera de Sacramento s/n, E-04120 La Cañada de San Urbano, Almería, Spain; E-Mails: emerlo@ual.es (M.E.M.); jmota@ual.es (J.F.M.); esanchez@ual.es (E.S.-S.); 2 Desertification Research Center (CIDE, CSIC-UV-GV), Carretera de Moncada-Náquera Km 4.5, E-46113 Moncada, Valencia, Spain; E-Mail: j.gabriel.segarra@uv.es

**Keywords:** Brassicaceae, conservation genetics, genetic diversity, gypsophile, next-generation sequencing, SSR

## Abstract

Polymorphic microsatellite markers were developed for the Ibero-North African, strict gypsophyte *Lepidium subulatum* to unravel the effects of habitat fragmentation in levels of genetic diversity, genetic structure and gene flow among its populations. Using 454 pyrosequencing 12 microsatellite loci including di- and tri-nucleotide repeats were characterized in *L. subulatum*. They amplified a total of 80 alleles (2–12 alleles per locus) in a sample of 35 individuals of *L. subulatum*, showing relatively high levels of genetic diversity, *H*_O_ = 0.645, *H*_E_ = 0.627. Cross-species transferability of all 12 loci was successful for the Iberian endemics *Lepidium cardamines*, *Lepidium stylatum*, and the widespread, *Lepidium graminifolium* and one species each of two related genera, *Cardaria draba* and *Coronopus didymus*. These microsatellite primers will be useful to investigate genetic diversity, population structure and to address conservation genetics in species of *Lepidium*.

## 1. Introduction

**Figure 1:**
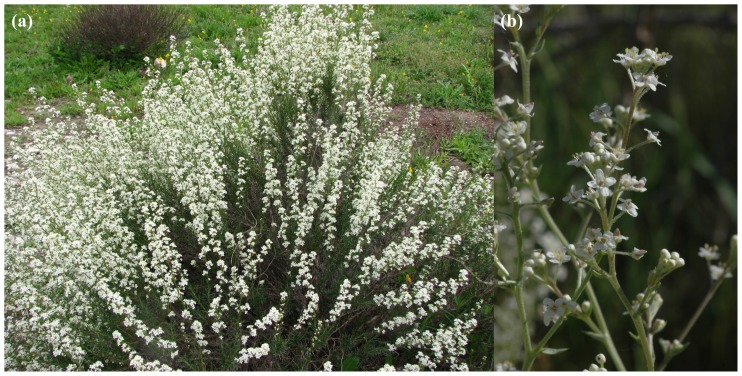
(**a**) General habit of *Lepidium subulatum*; (**b**) Detail of inflorescence.

*Lepidium* L. (Brassicaceae) is a cosmopolitan genus that includes about 175 species of annual to short-lived perennial herbs that inhabit predominantly ruderal habitats, shrublands and rock crevices on cliffs, primarily in temperate and subtropical regions [1]. Several species are adapted to more xeric, steppe habitats, such as the Ibero-North African endemic *Lepidium subulatum* L. (Sect. *Dileptium* (Raf.) DC.). This perennial gypsophyte (Figure 1) is diploid with 2*n* = 2*x* = 16 chromosomes [2]. This species is ecologically specialized and occurs exclusively in gypsum habitats [3]. Iberian gypsum outcrops show a naturally fragmented and scattered distribution on the eastern half of the Iberian Peninsula. They include a highly diverse and specialized flora including a large number of narrow endemics. However, such habitats have been subjected to diverse human-induced degradation because of their exploitation for gypsum extraction, and the establishment of landfills because of their comparatively lower agricultural productivity. Therefore, they have been identified as potentially sensitive areas for loss of biodiversity and of priority in conservation concerns.

Our primary goal is to investigate the effects of habitat fragmentation on the population genetics of selected species from Iberian gypsum outcrops. Given their fragmented distribution and the diverse degree of geographical connectivity and geographical extension, such areas can be considered as true ecological islands [4]. This is because of the ecological selectivity of gypsum soils resulting in an ecologically specialized flora that is unable to thrive in the surrounding areas with different substrates [4]. Thus, investigation of widespread gypsophytes such as *L. subulatum* could be used to exemplify general population biology and evolutionary dynamics of these ecologically selective habitats [5]. In addition, information on its population genetics and structure could be used to assist restoration of altered habitats (*i.e.*, abandoned gypsum quarries) with *L. subulatum*. For this purpose we have conducted a microsatellite characterisation in *L. subulatum* in order to investigate the effects of habitat fragmentation on genetic diversity, population structure and levels of gene flow among populations. We will perform genotypic analysis of individuals based on this set of nuclear microsatellite loci to (i) interpret historical and ecological processes affecting the gypsum habitats, and (ii) to propose relevant genetic and ecological units for conservation detected among the fragmented landscape. This study will further provide a valuable molecular tool for addressing strategies for the conservation of the biodiversity of gypsum habitats and for promoting population restoration of altered areas with suitable genotypes.

## 2. Results and Discussion

### 2.1. Polymorphism of Microsatellite Markers in *Lepidium subulatum*

Twelve loci with the best primer scores were selected from a shotgun 454-sequenced genomic library to investigate levels of genetic diversity at microsatellite loci in *L. subulatum* (Table 1).

The 12 polymorphic microsatellite loci detected a total of 80 different SSR alleles in the 35 individuals analysed of *L. subulatum*. The number of alleles ranged from a minimum of two alleles for locus Lsub09 to a maximum of 12 alleles for loci Lsub02 and Lsub04 and the mean number of alleles per locus was of 6.67 (Table 2). Observed heterozygosities ranged from *H*_O_ = 0.086 (locus Lsub09) to *H*_O_ = 0.914 (locus Lsub03) and expected heterozygosities ranged from *H*_E_ = 0.083 (locus Lsub09) to *H*_E_ = 0.881 (locus Lsub04). None of the 12 loci showed significant heterozygote deficiency, with an overall non-significant inbreeding coefficient value of *F*_IS_ = −0.030 (Table 2).

### 2.2. Cross Transferability of Microsatellite Markers to Other Lepidieae

All 12 microsatellite loci were transferable to all assayed species (Table 2). All loci were polymorphic in the Iberian endemic gypsophyte, *Lepidium cardamines* (Sect. *Dileptium*). 8 and 10 of the 12 loci were polymorphic in the high mountain, Iberian, narrow endemic *Lepidium stylatum* (Sect. *Lepia* (Desv.) DC.), and in the widespread ruderal, *L. graminifolium* (Sect. *Lepidium*), respectively. However, in the latter species individual genotypes could not be reliably scored from the amplification patterns with individuals showing up to five amplified bands per individual (Table 2). Ten and eight loci were polymorphic in *C. draba* and *C. didymus* respectively, with amplification patterns revealing up to six bands per individual in both taxa (Table 2). Thus, the multiple banding patterns observed at each locus supported the polyploid status of *Lepidium graminifolium* (2*n* = 6*x* = 48 [2]), *C. draba* (2*n* = 8*x* = 64 [6]) and *C. didymus* (2*n* = 4*x* = 32).

In the analysed polyploid species banding patterns were consistent with the aforementioned ploidy levels. Up to four bands per individual were amplified in the tetraploid *C. didymus*, and up to six bands were amplified in the octoploid *C. draba* (Table 2). In this latter species number of bands did not reach the expected maximum of eight bands per individual, most likely because the loci were not variable enough in the analysed population. A similar result was obtained in the hexaploid *L. gramminifolium* with up to five bands amplified per individual. Interestingly, in this species six out of 10 polymorphic loci showed fixed heterozygous profiles with individuals showing consistently a minimum of two amplified bands. This may indicate an allopolyploid hybrid origin for the species [7,8].

## 3. Experimental Section

### 3.1. Isolation of Microsatellite Markers

Five hundred nanograms of total DNA were extracted from silica-gel dried young leaves of a pool of individuals using the DNeasy plant minikit (Qiagen, Barcelona, Spain) following the manufacturer’s instructions, and eluted in 130 μL TE. This DNA was used to construct 454 genomic libraries by the sequencing service from the University of Valencia (SCSIE, Spain) and to conduct shotgun sequencing on a GS Junior 454 sequencer (Roche, Barcelona, Spain). We obtained 119,732 reads with an average read length of 465.61 bp and a total amount of 55,748,981 bases that were trimmed of adaptor and low-quality regions and assembled into contigs using GS De Novo Assembler implemented in Newbler 2.5p1 (Roche, Madrid, Spain), using default parameters (e.g., 40 bases minimum overlap and 100 bases minimum contig length). Generated contigs and unique reads not assigned to contigs were subjected to BLAST analysis and those matching organellar (chloroplast or mitochondria) sequences were discarded.

We screened all 8305 unique reads and non organellar contigs with iQDD v. 1.3.0.0 software [9]. We set the script to identify all possible di-, tri, tetra-, penta- and hexanucleotide repeats with a minimum of 4 repeat units, and compound repeats. Mononucleotide repeats were not considered. A total of 969 reads containing microsatellites were found in the 8305 screened reads, consisting of 623 dinucleotide, 143 trinucleotide, three tetranuleotides, one pentanucleotide, one hexanucleotide and 198 compound repeats. The program was allowed for direct primer design using PRIMER3 [10] by searching for microsatellite repeats and primer annealing sites in the flanking regions. After discarding reads with too short flanking sequences, primers were successfully designed for 652 reads. However, 405 of these (62.12%) corresponded to AT/TA repeats and were not considered because of their self-complementarity within DNA strains which could make them are more prone to amplification artefacts [11]. Of the 247 remaining loci, 21 dinucleotide and 17 trinucleotide microsatellite loci had more than seven repeat units. Twelve loci out of the 38 showing the best primer scores were selected for PCR amplification and as all of them produced clear amplicons of the expected size in 2% agarose gels they were subsequently selected for analysis on automated sequencers (Table 1). Forward primers were labelled with 6FAM, NED, PET or VIC fluorescent dyes from Applied Biosystems for automated electrophoresis (Table 1). PCR amplifications were performed in a 20 μL mix containing 1× *Taq* Buffer (Biotools, Madrid, Spain), 2 mM MgCl_2_, 0.4 mM of each dNTP, 5 pmol each of the labelled (forward) and unlabelled (reverse) primers, 1 U of *Taq* polymerase (Biotools) and 20 ng of template DNA. The PCR program consisted of one step of 4 min at 94 °C followed by 39 cycles each of 1 min at 94 °C, 1 min at 55 °C for annealing, and 1 min at 72 °C, and a final extension step of 7 min at 72 °C. The products were run on an ABI 3730XL automated sequencer (Applied Biosystems) using LIZ500 as the internal lane size standard, and the amplified fragment lengths were assigned to allelic sizes with GENEMARKER v. 1.85 software (SoftGenetics, State College, PA, USA). After an initial screening of individuals, all 12 loci showed consistent amplification patterns and polymorphisms and were subsequently used for genotyping the entire set of samples.

### 3.2. Data Analysis

Genotypic data were obtained for 35 individuals from one population of *L. subulatum* (Table 3) for 12 microsatellite loci (Tables 1 and 2).

Number of alleles (*N*_A_), observed (*H*_O_) and unbiased expected (*H*_E_) heterozygosities [12] were calculated with GENETIX v. 4.05 [13]. Inbreeding coefficients (*F*_IS_) and deviations from Hardy-Weinberg equilibrium and linkage disequilibrium between pairs of microsatellite loci, using 1000 permutations, were calculated using GENEPOP v.4.1.4 software [14]. None of the 66 pairwise comparisons between loci showed significant linkage disequilibrium (*p* < 0.05).

Cross-species transferability was assayed in one population each of three other species of *Lepidium* (*L. cardamines* L., *L. graminifolium* L., and *L. stylatum* Lag. & Rodr., Table 3), and one species each of the closely related genera *Cardaria* Desv. (*C. draba* L.), and *Coronopus* Zinn (*C. didymus* L., Table 3).

## 4. Conclusions

The results obtained in this exploratory analysis of the genetic diversity in *L. subulatum* with 12 novel nuclear polymorphic microsatellite loci support their use for conducting population genetics and to investigate the effects of habitat fragmentation on gene flow among populations and population genetic structure of this endemic gypsophyte. The successful cross-transferability of all these markers to *L. cardamines* and *L. stylatum* further expands their usefulness to address similar questions in these two restricted Iberian endemics. The genetic information rendered by these microsatellites will enable more efficient conservation programs for these species. The cross-transferability to other *Lepidieae* potentially broadens their applicability to a large number of taxa in *Lepidieae*, including polyploids.

## Figures and Tables

**Table 1:** Characteristics of 12 polymorphic microsatellite loci developed for *Lepidium subulatum*. For each locus the primer pair sequences, repeat motif, size of the original fragment (bp), and Genbank accession numbers are shown. 6FAM, NED, PET, and VIC are fluorescent dyes from Applied Biosystems (Madrid, Spain).

Locus	Primer sequence (5′→3′)	Repeat motif	Size	GenBank accession No.
**Lsub01**	F: 6FAM-CTTTCTCGCTGAGCTGTCAA R: TTGTCTCTGCCGAAATCCAT	(GA)_12_	201	JQ663929
**Lsub02**	F: VIC-GGATTTAATTCGTGGACAGCA R: CACCGACTACTCCGATCCTC	(AG)_9_	209	JQ663930
**Lsub03**	F: PET-CAAATGAAAGCAGATCAAGCA R: TGGATCAATTTCCTGTTGGA	(AG)_12_	182	JQ663931
**Lsub04**	F: NED-TCCATTGATATTCCGAGCAA R: GGGTTACGTGATTTAGGGAACA	(TCA)_22_	202	JQ663932
**Lsub05**	F: 6FAM-GGGTTTGTCCCACAAGAAGA R: CAGGTCAATCGCGTGTTCTA	(GA)_9_	293	JQ663933
**Lsub07**	F: NED-CCAATCAATACCATCTCCCAAG R: TGTCGTTAGAATCTTGCTGAATGT	(TG)_10_	174	JQ663934
**Lsub08**	F: PET-GCCAACGTACAACGGAGAAT R: ATCCGATTTCGTCACTCTGC	(GA)_10_	184	JQ663935
**Lsub09**	F: VIC-AATGGTGGGCTCGGATTTA R: CCTTTGTTCGATTCCCAATG	(TC)_8_	171	JX399866
**Lsub10**	F: NED-TGGTGGAGAGGACAAAGGAT R: TCAACGTAAAGCAACCCAAA	(GA)_8_	273	JX399867
**Lsub11**	F: 6FAM-ACTCCGATAAATTGGGCATC R: CAAATCTCCATTTCTCGACCA	(AG)_8_	182	JX399868
**Lsub12**	F: VIC-AGCTGGAGATCCGAAGAACA R: TCCATTGAAACCTCAACGTG	(GAA)_9_	181	JQ663936
**Lsub13**	F: PET-GCCGAATAAGAGGGAGTTGC R: CGCCCACTCCTAACTCTCAC	(AG)_8_	152	JX399869

**Table 2:** Results of initial primer screening for 12 polymorphic loci in three diploid and three polyploid species of *Lepidieae*. For each locus in the diploid species, allele range (*A*_r_), number of alleles (*N*_A_), observed (*H*_O_) and expected (*H*_E_) heterozygosities and *F*_IS_ values are reported. For polyploid species allele range (*A*_r_), number of alleles (*N*_A_), and range of number of alleles per individual (*N*_AI_) are reported. Ploidy levels for polyploidy species are indicated in brackets.

Diploids																Polyploids								
	
*Lepidium subulatum*						*Lepidium cardamines*					*Lepidium stylatum*					*Lepidium graminifolium* (2*n* = 6*x* = 48)			*Cardaria draba* (2*n* = 8*x* = 64)			*Coronopus didymus* (2*n* = 4*x* = 32)		
					
Locus	*A*_r_	*N*_A_	*H*_O_	*H*_E_	*F*_IS_	*A*_r_	*N*_A_	*H*_O_	*H*_E_	*F*_IS_	*A*_r_	*N*_A_	*H*_O_	*H*_E_	*F*_IS_	*A*_r_	*N*_A_	*N*_AI_	*A*_r_	*N*_A_	*N*_AI_	*A*_r_	*N*_A_	*N*_AI_
					
Lsub01	187–223	10	0.886	0.821	−0.079	189–195	4	0.500	0.480	−0.041	187–205	4	0.615	0.612	−0.005	183–191	4	2–4	185	1	1	179–201	8	2–4
Lsub02	205–241	12	0.800	0.790	−0.013	193–253	10	0.800	0.829	+0.035 *	211–221	2	0.462	0.442	−0.044	199–203	3	2–3	225–233	2	2	231	1	1
Lsub03	170–196	10	0.914	0.848	−0.078	174–182	4	0.500	0.678	+0.262	220	1	0.000	0.000	-	186–190	3	2–3	204–220	3	1–3	164	1	1
Lsub04	151–196	12	0.857	0.881	+0.027	193–253	12	0.800	0.813	+0.016 *	166–175	4	0.750	0.625	−0.200	151–259	15	1–4	154–265	14	1–6	151–198	14	1–4
Lsub05	191–299	5	0.686	0.663	−0.034	299–341	10	0.800	0.861	+0.070 **	277–294	7	0.615	0.837	+0.264	259–301	9	2–5	277–279	2	2	229–257	2	2
Lsub07	166–176	6	0.743	0.698	−0.064	164–170	4	0.750	0.749	−0.002	168–174	4	0.700	0.689	−0.016	170–172	2	1–2	168–174	4	1–3	168–174	4	1–3
Lsub08	184–198	8	0.771	0.778	+0.009	178–188	5	0.700	0.733	+0.045	184	1	0.000	0.000	-	176	1	1	166–182	2	2	182	1	1
Lsub09	171–173	2	0.086	0.083	−0.030	177–193	7	1.000	0.828	−0.208	165–167	2	0.692	0.462	−0.500	159–169	5	2–4	135–163	2	2	169	1	1
Lsub10	271–277	4	0.657	0.668	+0.016	273–305	6	0.625	0.791	+0.211 **	281–291	3	0.385	0.590	+0.348	259–273	3	2–3	271–281	5	1–4	271–277	4	1–4
Lsub11	180–184	3	0.400	0.340	−0.175	176–178	2	0.000	0.100	+1.000 *	178	1	0.000	0.000	-	180–184	2	1–2	178	1	1	178–182	2	1–2
Lsub12	169–178	5	0.657	0.696	+0.056	166–193	4	0.600	0.666	+0.099	178–190	4	0.615	0.692	+0.111	145	1	1	169–178	3	3	166–184	2	1–2
Lsub13	152–156	3	0.286	0.255	−0.122	154–158	2	0.500	0.382	−0.310	154	1	0.000	0.000	-	152–156	3	1–2	152–154	2	1–2	148–154	4	1–3
Mean		6.67	0.645 ± 0.257	0.627 ± 0.257	−0.030		5.83	0.631 ± 0.251	0.658 ± 0.229	+0.039 ***		2.83	0.403 ± 0.314	0.413 ± 0.321	+0.047		4.25			3.42			3.67	

* *p* < 0.05;

** *p* < 0.01;

*** *p* < 0.001.

**Table 3:** Voucher information for taxa used in this study.

Taxon	Locality	Geographical coordinates	Altitude (m)	Collectors	Voucher	*N* (sample size)
*Lepidium subulatum*	Granada: Venta del Peral	37°33′10.6″N, 02°37′39.8″W	745	*E. Salmerón*	HUAL 24484	35
*Lepidium cardamines*	Cuenca: El Pedernoso, llanos de la Motilla	39°29′45″N, 02°46′42″W	710	*G. Mateo & V.J. Arán*	MA 599153	20
*Lepidium graminifolium*	Valencia: Gardens next to “Turia” Metro station	39°28′38.6″N, 00°23′23.2″W	17.2	*I. Martínez-Nieto*	HUAL 24482	30
*Lepidium stylatum*	Granada: Sierra Nevada, Laguna de Aguas Verdes	37°02′54″N, 3°22′05″W	3060	*A.B. Robles & P. Sánchez*	GDAC17379	13
*Cardaria draba*	Valencia: Sagunto, Marjal del Moro	39°37′34.0″N, 00°16′02.6″W	0.5	*I. Martínez-Nieto*, *J.G. Segarra-Moragues & M.J. Gil-López*	HUAL 24483, 24488	30
*Coronopus didymus*	Valencia: Moncada, pr. Instituto Valenciano de Investigaciones Agrarias (IVIA)	39°35′20.1″N, 00°23′42.4″W	66.5	*I. Martínez-Nieto & J.G. Segarra-Moragues*	HUAL 24481	30
